# Magnetic entropy table-like shape and enhancement of refrigerant capacity in La_1.4_Ca_1.6_Mn_2_O_7_–La_1.3_Eu_0.1_Ca_1.6_Mn_2_O_7_ composite

**DOI:** 10.1039/c9ra00984a

**Published:** 2019-05-14

**Authors:** R. M'nassri, Muaffaq M. Nofal, P. de Rango, N. Chniba-Boudjada

**Affiliations:** Unité de recherche Matériaux Avancés et Nanotechnologies (URMAN), Institut Supérieur des Sciences Appliquées et de Technologie de Kasserine, Kairouan University BP 471 Kasserine 1200 Tunisia rafik_mnassri@yahoo.fr; Department of Mathematics and General Sciences, Prince Sultan University P. O. Box 66833 Riyadh 11586 Saudi Arabia; Institut NEEL BP 166 38042 Grenoble Cedex 09 France

## Abstract

In this work, we have investigated the structural, magnetic and magnetocaloric properties of La_1.4_Ca_1.6_Mn_2_O_7_ (A) and La_1.3_Eu_0.1_Ca_1.6_Mn_2_O_7_ (B) oxides. These compounds are synthesized by a solid-state reaction route and indexed with respect to Sr_3_Ti_2_O_7_-type perovskite with the *I*4/*mmm* space group. The substitution of La by 10% Eu enhances the value of magnetization and reduces the Curie temperature (*T*_C_). It is also shown that these compounds undergo a first-order ferromagnetic–paramagnetic phase transition around their respective *T*_C_. The investigated samples show large magnetic entropy change (Δ*S*_M_) produced by the sharp change of magnetization at their Curie temperatures. An asymmetric broadening of the maximum of Δ*S*_M_ with increasing field is observed in both samples. This behaviour is due to the presence of metamagnetic transition. The Δ*S*_M_(*T*) is calculated for A_*x*_/B_1−*x*_ composites with 0 ≤ *x* ≤ 1. The optimum Δ*S*_M_(*T*) of the composite with *x* = 0.48 approaches a nearly constant value showing a table-like behaviour under 5 T. To test these calculations experimentally, the composite with nominal composition A_0.48_/B_0.52_ is prepared by mixing both individual samples A and B. Magnetic measurements show that the composite exhibits two successive magnetic transitions and possesses a large MCE characterized by two Δ*S*_M_(*T*) peaks. A table-like magnetocaloric effect is observed and the result is found to be in good agreement with the calculations. The obtained Δ*S*_M_(*T*) is ≈4.07 J kg^−1^ K^−1^ in a field change of 0–5 T in a wide temperature span over Δ*T*_FWHM_ ∼ 68.17 K, resulting in a large refrigerant capacity value of ≈232.85 J kg^−1^. The MCE in the A_0.48_/B_0.52_ has demonstrated that the use of composite increases the efficiency of magnetic cooling with *μ*_0_*H* = 5 T by 23.16%. The large Δ*T*_FWHM_ and RC values together with the table-like (−Δ*S*_M_)^max^ feature suggest that the A_0.48_/B_0.52_ composite can meet the requirements of several magnetic cooling composites based on the Ericsson-cycle. In addition, we show that the magnetic field dependence of MCE enables a clear analysis of the order of phase transition. The exponent *N* presents a maximum of *N* > 2 for A, B and A_0.48_/B_0.52_ samples confirming a first-order paramagnetic–ferromagnetic transition according to the quantitative criterion. The negative slope observed in the Arrott plots of the three compounds corroborates this criterion.

## Abstract 2

In this work, we have investigated the structural, magnetic and magnetocaloric properties of La_1.4_Ca_1.6_Mn_2_O_7_ (A) and La_1.3_Eu_0.1_Ca_1.6_Mn_2_O_7_ (B) oxides.

## Graphical abstract

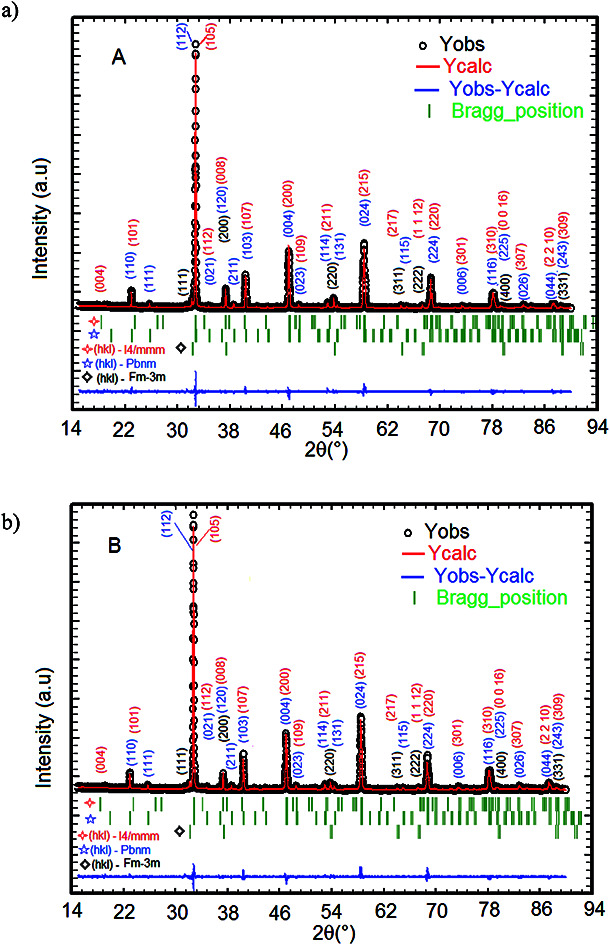

## 1. Introduction

Manganese oxides exhibiting colossal magnetoresistance (CMR) and large magnetocaloric effect (MCE) are a very hot topic in materials science, not only because of the interest they have generated in basic research but also for their potential technological applications in spintronics and magnetic refrigeration (MR).^1,2^ In addition, their adjustable phase transition temperatures, low-price and wear and corrosion resistance provide an additional advantage for the choice of manganese oxides as magnetic refrigerant materials for designing a “green” cooling refrigerator.^3–5^ Magnetic refrigerators are considered an ecologically friendly technology because of several advantages they have compared to traditional refrigerators. High efficiency, small volume, and being free of harmful gas leakage are among these advantages.^6^ The latter machines work on the principle of the MCE,^7,8^ which describes the adiabatic temperature change of magnetic substance produced by the magnetic entropy change (Δ*S*_M_) upon magnetization and demagnetization.^9^ When magnetics stimulus in an adiabatic process is applied, the entropy of the spin subsystem is diminished and the transfer of energy to the lattice produces heating of the magnetic substance. Conversely, removing magnetics stimulus of the substance causes it to cool down.^10,11^ The exploration of new refrigerant materials with large MCE at both ambient and cryogenic temperatures is strongly desired and is vital in accelerating the progress of magnetic cooling technology. Nevertheless, large MCE and negligible thermal and magnetic-field hysteresis losses are required for MR. Considering the various requirements for applying magnetic cooling, La_2−2*x*_Ca_1+2*x*_Mn_2_O_7_ Ruddlesden–Popper phases (*n* = 2) possess high magnetic moments and have a giant magnetocaloric effect. A giant peak value of Δ*S*_M_ (16.8 J kg^−1^ K^−1^) originating from the abrupt change of magnetization observed at 5 T in La_1.4_Ca_1.6_Mn_2_O_7_ system.^12^ This value is mostly close to that of systems undergoing a first order magnetic phase transition (FOMT) such as Gd_5_GeSi_2_ (18.5 J kg^−1^ K^−1^) and MnFeP_1−*x*_As_*x*_ (18 J kg^−1^ K^−1^) alloys under the same field change.^13^ Generally, the Δ*S*_M_ is adopted as an important index to demonstrate the refrigerant ability. Moreover, for a sample exhibiting FOMT, the value of Δ*S*_M_ is highest near the magnetic transition temperature and falls rapidly with temperature, making its usage limited over a narrow temperature range.^10^ Even though the change in magnetic entropy is large in such type of materials, they exhibit large thermal and field hysteresis on variation of magnetization with temperature and magnetic field, respectively. However, a considerable refrigerant capacity (RC), besides a giant peak entropy change, is also essential to obtain an excellent refrigeration efficiency. In this context, FOMT compounds do not seem to be the best choices, as their large hysteresis losses and limited temperature spans lead to significant decreases in refrigerant capacities. From the practical application point of view, materials with a large MCE over a broad temperature range are desired. However, it is therefore interesting to search for new FOMT materials with low-level hysteresis, high performance and excellent functional stability. Among the presently known MCE materials with a first-order magnetic transition (FOMT) the La_1.4_Ca_1.6_Mn_2_O_7_ compounds fulfill most of the requirements for practical applications of magnetic refrigeration. First, it has a limited thermal hysteresis at the FOMT.^14^ Second, it is easy to tune the operating temperature by varying the La/Ca ratio or by substituting the Mn ion by various transition metal. Furthermore, the composition of this compound is low priced, have good chemical stability, easy to prepare, and does not contain any toxic or expensive elements such as arsenic and germanium, respectively.

It is well known that the structure of La_1.4_Ca_1.6_Mn_2_O_7_ is constructed from ferromagnetic metal bilayer slices of MnO_2_ sheets taken from the cubic perovskite, each slice being separated by a nonmagnetic insulating spacer layer which serves to isolate the bilayers (La, Ca)_2_O_2_ stacked along the *c*-axis. The anisotropy and the reduced dimensionality of these compounds play a crucial role in their special properties different from those shown by the cubic perovskites.^15–17^ Basically, the simultaneous ferromagnetic and metallic states observed in the Mn-based perovskite are explained using the double exchange mechanism (DE) caused by charge disproportionation.^18–20^ The DE interaction in the Mn–O–Mn network in the case of bilayer manganite is expected to be much weaker along the stacking *c*-axis direction because of the intervening rock salt layer that disrupts the interaction between the [MnO_2_] layers. Members of this perovskite family are very responsive to small changes in composition and structure because of their layered structure. An inherent anisotropy modifies the thermomagnetic properties of the layered materials.

In the context of magnetic cooling, the La_1.4_Ca_1.6_Mn_2_O_7_ compound shows an abrupt change in the magnetization ((∂*M*/∂*T*)_H_) and illustrates his magnetic entropy (Δ*S*_M_(*T*)) with particularly peak at Curie temperature. However, in regenerative Ericsson cycle, the entropy change of the refrigerant Δ*S*_M_(*T*) should be constant (table-like MCE) over the operating temperature range of about 30 K. For this, there are number of publications^21–23^ in which the authors have proposed different solutions to improve the cooling capacity at larger spans. Therefore, a solution is to work with a multiphase or sandwich materials. These materials extend the temperature range in which the magnetic entropy changes significantly increase the possibility of improving performance through layering. Another simple way that increases the efficiency of magnetic cooling of bilayer manganites is the creation of the composite by a succession of magnetocaloric refrigerant samples with similar values of Δ*S*_M_ and refrigerant capacity (RC).^24,25^ In this work, an optimum molar fraction of La_1.4_Ca_1.6_Mn_2_O_7_ (A) and La_1.3_Eu_0.1_Ca_1.6_Mn_2_O_7_ (B) is determined for the assembling of a composite to be used as refrigerant material in solid-state magnetic cooling. A physical mixture of La_1.4_Ca_1.6_Mn_2_O_7_ and La_1.3_Eu_0.1_Ca_1.6_Mn_2_O_7_ is introduced to extend the operating temperature window because *T*_C_ of La_1.4_Ca_1.6_Mn_2_O_7_ can be modified by small addition of Eu in La site. In addition, to extend the range of refrigeration, a composite magnetic refrigerant can be also used to increase or to optimize the refrigeration capacity (RC). This represents approximately the total thermal energy transferred from the hot to cold reservoirs over the active temperature range. Therefore, it was demonstrated that mixing of La_1.4_Ca_1.6_Mn_2_O_7_ (A) and La_1.3_Eu_0.1_Ca_1.6_Mn_2_O_7_ (B) provides an extra material design tool such that the optimal magnetic refrigerant material can be developed for a specific temperature range. The experimental results agree well with those calculated and discussed in the framework of an optimum regeneration Ericsson cycle. The MCE and RC of a prepared composite have been compared with those of individual bilayer manganites.

## 2. Experimental details

In this work, standard ceramic process is used to prepare two samples: La_1.4_Ca_1.6_Mn_2_O_7_ (sample A) and La_1.3_Eu_0.1_Ca_1.6_Mn_2_O_7_ (sample B). Well-grounded stoichiometric mixture of La_2_O_3_, MnO_2_, CaCO_3_ and Eu_2_O_3_ with a purity of (99.9%) is prepared. These contents are mixed and grounded, then sintered for 12 h at 1200 °C. Subsequently pressed into pellets, which and sintered again for 12 h at 1200 °C. After grinding, the annealed powders are then pressed into disks and sintered at 1400 °C for 12 h with intermittent grinding and slow cooling in a furnace. The obtained disk-shaped samples are well grounded again, then pelletized and sintered at 1400 °C for 24 h. Finally, the sintered ceramic samples are slowly cooled to room temperature in air. As the sample have been elaborated in air, it is consequently stoichiometric in oxygen.^26,27^ The composite sample is made by thoroughly mixing 48% : 52% (by weight) of polycrystalline powders of La_1.4_Ca_1.6_Mn_2_O_7_ and La_1.3_Eu_0.1_Ca_1.6_Mn_2_O_7_ in an agate mortar for 30 min. The obtained compound will be referred as A_0.48_/B_0.52_. The samples are characterized using X-ray powder-diffraction measurements at room temperature in the 2*θ* range of 20° to 80° with CuKα radiation (*λ* = 1.5406 Å). The structural parameters are refined by Rietveld's profile-fitting method using Fullprof software. The temperature-dependence and the magnetic-field-dependence of the magnetization, *M*(*T*) and *M*(*μ*_0_*H*), are performed around the Curie temperature (*T*_C_) using vibrating sample magnetometer developed at NEEL Institute.

## 3. Results and discussions

**Fig. 1:**
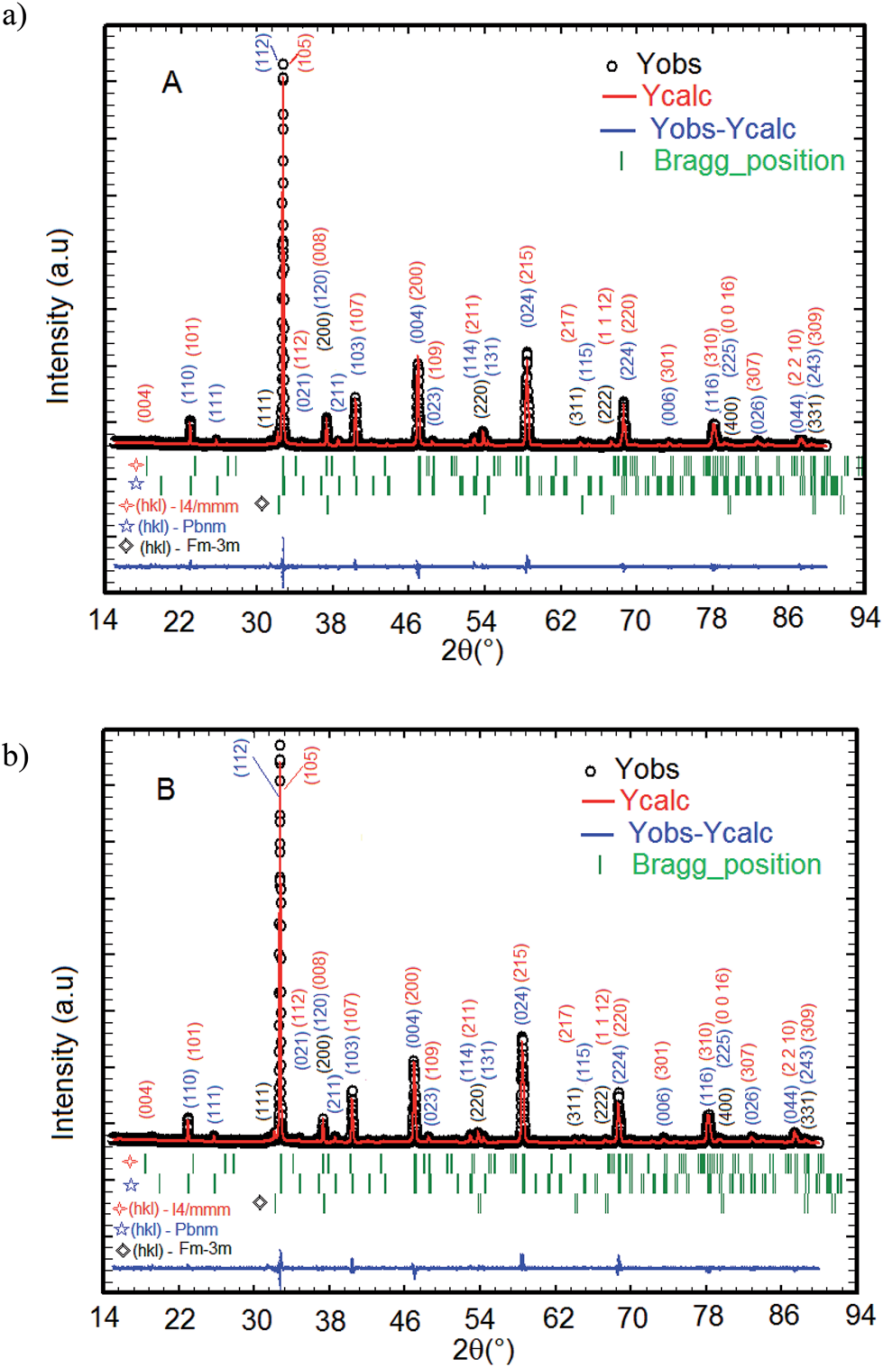
The XRD patterns for A and B samples performed at room temperature. (a) La_1.4_Ca_1.6_Mn_2_O_7_ and (b) La_1.3_Eu_0.1_Ca_1.6_Mn_2_O_7_.

The XRD patterns of La_1.4_Ca_1.6_Mn_2_O_7_ (A) and La_1.3_Eu_0.1_Ca_1.6_Mn_2_O_7_ (B) samples registered at 300 K and the structural refinement patterns showing the observed, calculated, and difference profiles for the final fit for the A and B samples, are depicted in Fig. 1(a) and (b). The phase identification and structural analysis of both samples are performed using the FullProf software.^28,29^ It is found that all diffraction peaks can be indexed with respect to Sr_3_Ti_2_O_7_-type perovskite with *I*4/*mmm* space group. As a La-bilayer-structured perovskite, these compounds are generally formed of the bilayers MnO_2_ (magnetic conducting layer) separated by a monolayer rock-salt-type (La, Ca)_2_O_2_ (non-magnetic insulating layer) along the *c* axis. Meanwhile, some small secondary phases attributed to the presence of CaO impurity with space group *Fm*3̄*m* and a fraction of with La_0.67_Ca_0.33_MnO_3_ type orthorhombic structure with space group *Pbnm* are observed in both samples. Both impurities are identified with X'Pert HighScore Plus software. For both compounds, the positions of the La_1.4_Ca_1.6_Mn_2_O_7_ for A sample (La_1.3_Eu_0.1_Ca_1.6_Mn_2_O_7_ for B sample), La_0.67_Ca_0.33_MnO_3_, and CaO peaks are denoted by the three sets of vertical bars (green color online), the top row corresponding to La_1.4_Sr_1.6_Mn_2_O_7_ (La_1.3_Eu_0.1_Ca_1.6_Mn_2_O_7_), *etc.* The Rietveld refinement of the XRD data indicates that the replacement of 10% lanthanum with europium in the system does not affect the tetragonal structure but causes a decrease in the cellular parameters *a* = *b*, *c* and the unit cell volume *V*. The lattice parameters of these compounds are found to be *a* = 3.870 Å, *c* = 19.302 Å and *V* = 289.059 Å^3^ for La_1.4_Ca_1.6_Mn_2_O_7_ (A) and *a* = 3.866 Å, *c* = 19.281 Å and *V* = 288.219 Å^3^ for La_1.3_Eu_0.1_Ca_1.6_Mn_2_O_7_ (B). The quality of the refinement is evaluated through the goodness of the fit indicator *χ*^2^, which is 1.32% for A sample and 1.43% for B sample. This confirms that the refinement is acceptable. The profile factor is found to be *R*_p_ = 19.3% (19.3%), weighed profile factor *R*_wp_ = 20.2% (20.6%) and Bragg *R*-factor *R*_Bragg_ = 7.57% (4.98%) for A sample (for B sample). The amounts of all phases present in the sample are quantified simultaneously using the Rietveld method. The phase quantification procedure involves the identification of major and minor phases. Here, quantitative phase analysis obtained by Rietveld refinement shows that the La_1.4_Ca_1.6_Mn_2_O_7_ (La_1.3_Eu_0.1_Ca_1.6_Mn_2_O_7_) is the dominant phase, constituting 89.7% (87.4%) of the weight. The La_0.67_Ca_0.33_MnO_3_ and the CaO phases account for only 6.1% (7.9%) and 4.2% (4.7%), respectively. The latter phase is frequently encountered after the final step of the synthesis of LaCa-bilayer manganites. Given the small concentration of the impurities, we assume that the secondary phase does not have any significant effect on the subsequent measurements of physical properties.

**Fig. 2:**
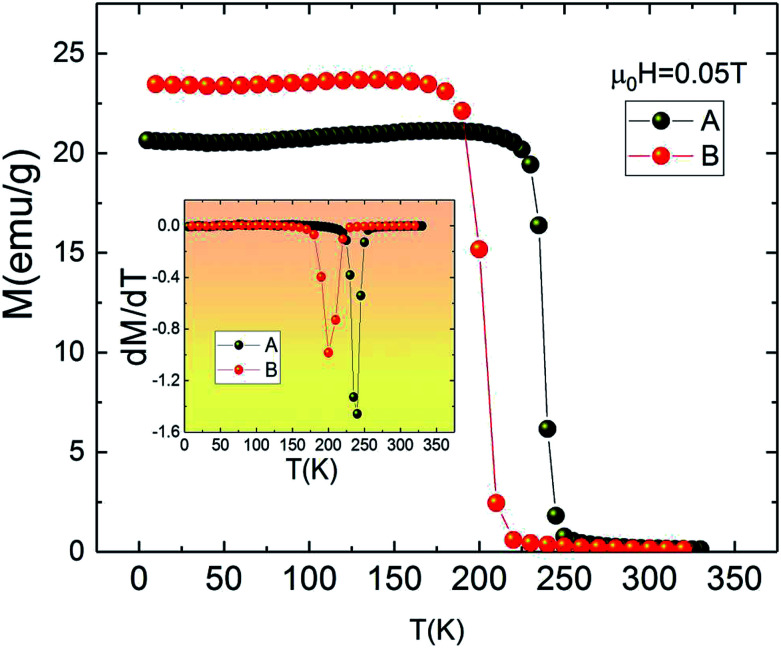
Magnetization measurements as a function of temperature for La_1.4_Ca_1.6_Mn_2_O_7_ and La_1.3_Eu_0.1_Ca_1.6_Mn_2_O_7_ samples under 0.05 T. Insets: d*M*/d*T* as a function of temperature.

Fig. 2 depicts the temperature dependence of magnetization *M*(*T*) for both A and B samples under an applied magnetic field of 0.05 T in field cooled mode (FC). It is clear from Fig. 2 that the *M*(*T*) curves do not reveal secondary magnetic phases, which in turn suggests that the presence of small amount of impurities does not have any significant effect on the thermomagnetic properties. The *M*(*T*) curves show rapid decrease of magnetization at Curie temperature which is the signature of the transition from ferromagnetic to paramagnetic state.

Furthermore, it can be seen from Fig. 2 that replacing La by Eu causes the value of *T*_C_ to shift from 240 K for La_1.4_Ca_1.6_Mn_2_O_7_ (A) to 200 K for La_1.3_Eu_0.1_Ca_1.6_Mn_2_O_7_ (B). The Curie temperature *T*_C_ is defined as the inflection point of d*M*/d*T* (see inset Fig. 2). It is clear that the pristine compound La_1.4_Ca_1.6_Mn_2_O_7_ is ferromagnetic below *T*_C_ = 240 K. This value is higher than *T*_C_ = 235 K given by ref. 30 and smaller than *T*_C_ = 270 K observed in the same sample provided by ref. 31. This dissimilarity may be explained by the sensitivity of Curie temperature to the preparation conditions and the temperature of sintering^32,33^ which leads to the conclusion that the preparation processes have enormous impacts on the performance of magnetic materials.

**Fig. 3:**
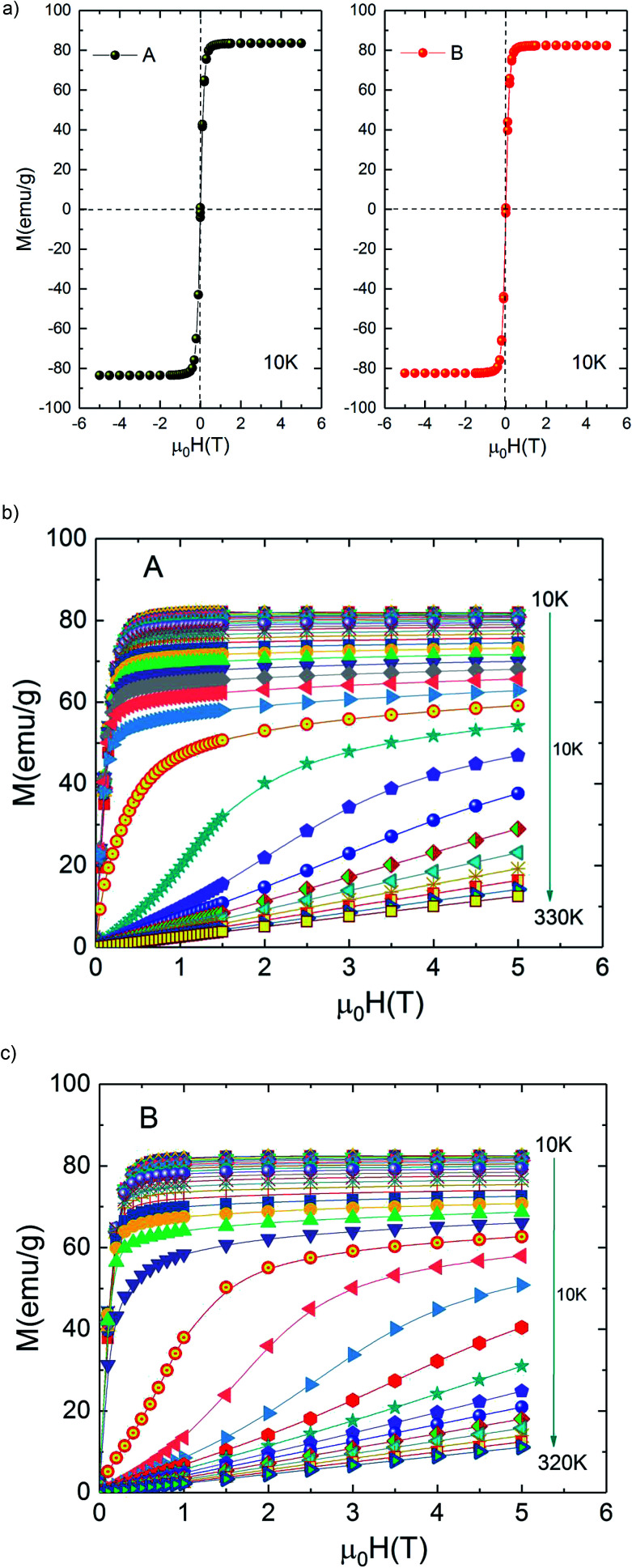
(a) Hysteresis loops for La_1.4_Ca_1.6_Mn_2_O_7_ and La_1.3_Eu_0.1_Ca_1.6_Mn_2_O_7_ samples at 10 K. Isothermal magnetization curves at various temperatures (b) La_1.4_Ca_1.6_Mn_2_O_7_ and (c) La_1.3_Eu_0.1_Ca_1.6_Mn_2_O_7_.

Fig. 3(a) shows the magnetic hysteresis loops of both A and B samples taken at 10 K. Both loops show nearly zero coercivity, high magnetization saturation and negligible hysteresis which means that A and B bilayer manganites exhibit perfect magnetic reversibility or soft ferromagnetic nature. These observed outstanding soft-magnetic properties are beneficial for the application as bulk magnetic refrigerants. Furthermore, it can be seen that they display scarcely any hysteresis loss, although the two compounds exhibit the nature of first-order phase transition. This point is very attractive for magnetic refrigeration.

Isothermal magnetization *M*(*μ*_0_*H*) curves are performed around transition temperature for each sample. Fig. 3(b) and (c) represents the recorded *M*(*μ*_0_*H*) curves of samples A and B over a wide range of the magnetic field ranging from 0 T to 5 T. At temperatures above 270 K for A sample (250 K for B sample) *M*(*μ*_0_*H*) curves show a linear behaviour as expected in the paramagnetic state. Below 220 K for A sample (190 K for B sample) *M*(*μ*_0_*H*) curves show an expected rapid increase at field values less than 0.4 T followed by the tendency to saturation at higher fields, which indicate the existence of a ferromagnetic state in the samples.

However, it can be clearly seen that the magnetization initially increases gradually with increasing *μ*_0_*H* for temperatures between 220 and 270 K for A sample (190 and 250 K for B sample). A sudden change appears above a critical magnetic field followed by a rapid increase of magnetization thus exhibiting an ‘S’ shaped *M*(*μ*_0_*H*) plot. This is a signature of a metamagnetic behaviour observed in the both samples. The latter phenomenon indicates the possibility of a large magnetic entropy change around Curie temperature. This point is very enticing for magnetic refrigeration. Motivated by our previous result of a large magnetocaloric effect (−Δ*S*_M_(*T*, *μ*_0_*H*) = 7.23 J kg^−1^ K^−1^ at 5 T (ref. 27)) observed in Pr_0.8_K_0.2_MnO_3_ sample which showed a similar metamagnetic behavior, we investigated the MCE in both aforementioned compounds.^26^

**Fig. 4:**
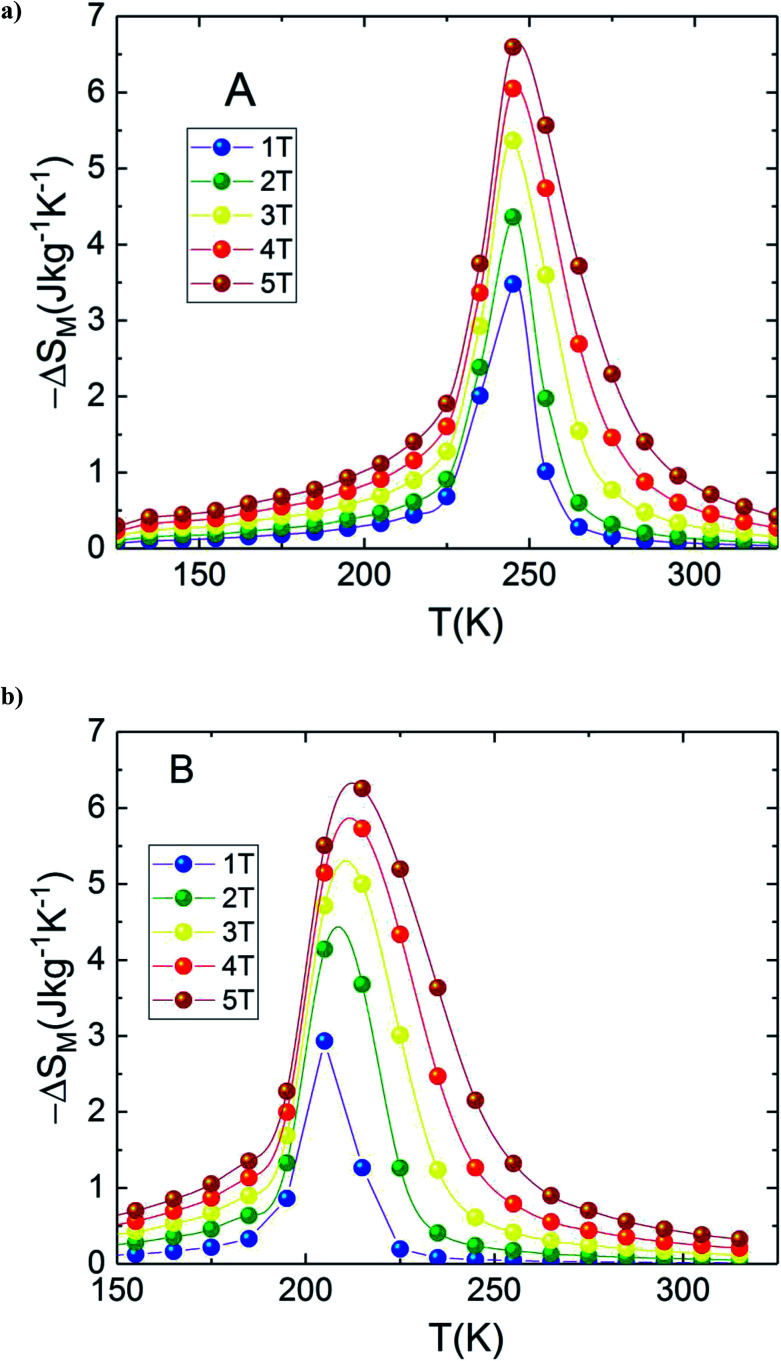
Magnetic entropy change, (Δ*S*_M_) *vs.* temperature for (a) La_1.4_Ca_1.6_Mn_2_O_7_ and (b) La_1.3_Eu_0.1_Ca_1.6_Mn_2_O_7_.

In the present work, it is interesting to evaluate the magnetocaloric effect of the A and B compounds. For this reason, we used the isothermal magnetisations measured at discrete temperatures to determine the MCE for each compound. Using Maxwell relation and magnetization curves (*M*–*μ*_0_*H*) we obtained the value of magnetic entropy changes Δ*S*_M_(*T*, *μ*_0_*H*) as a function of temperature in the magnetic field range of 0 to 5 T for both A and B samples. Fig. 4(a) and (b) depicts the behaviour of Δ*S*_M_(*T*, *μ*_0_*H*) for both compounds. The negative sign of the Δ*S*_M_(*T*, *μ*_0_*H*) seen in the latter figures is referred as the normal MCE and confirms the ferromagnetic nature of these samples.^34–36^ As one can see, the aforementioned materials illustrate significant values ​​of the magnetic entropy changes and show that the magnitudes of Δ*S*_M_ increases with an increase in the applied magnetic field. For *μ*_0_*H* = 5 T, the entropy change Δ*S*_M_ exhibits a maximum value of 6.6 J kg^−1^ K^−1^ around *T*_peak_ ∼ 245 K for A sample (6.25 J kg^−1^ K^−1^ around *T*_peak_ ∼ 215 K for B sample) and it decreases on either side. However, the magnitude of Δ*S*_M_ increases and the peak of Δ*S*_M_ becomes asymmetrical with the rise of magnetic field. While Δ*S*_M_ diminishes abruptly with lowering temperature below the peak, it gradually falls with the rise of temperature above the peak. We can also remark that Δ*S*_M_ curves for the both samples present higher peak values and are quite similar in the temperature range of Δ*T* = *T*_CA_ − *T*_CB_ ≈ 40 K. Due to the remarkable similarities in the results, the two materials provide an opportunity to manufacture a composite with high performance in the context of magnetic refrigeration.

**Fig. 5:**
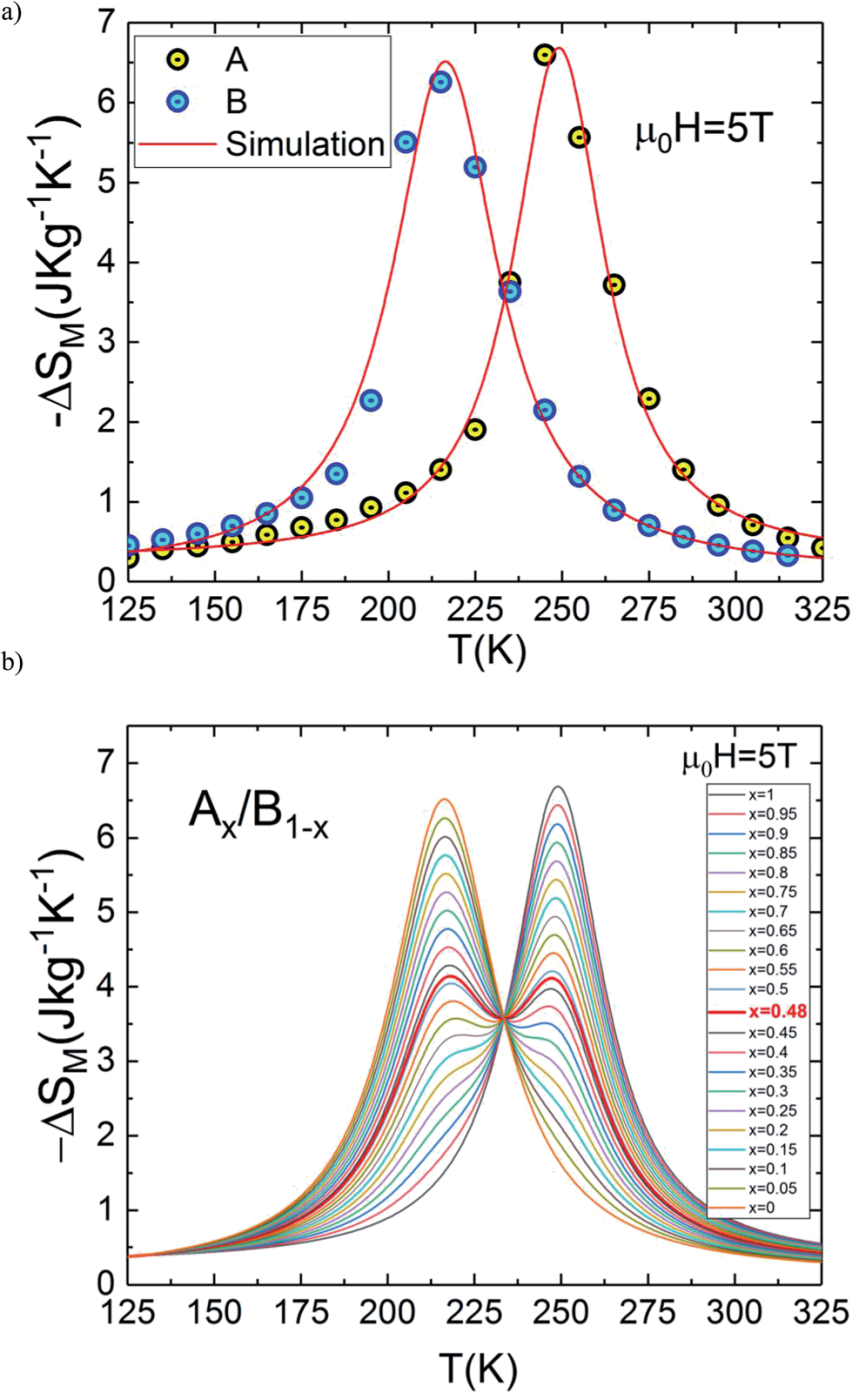
(a) Δ*S*_M_(*T*, *μ*_0_*H* = 5 T) for La_1.4_Ca_1.6_Mn_2_O_7_ and La_1.3_Eu_0.1_Ca_1.6_Mn_2_O_7_. (b) Δ*S*_M_(*T*) curves for the composite (La_1.4_Ca_1.6_Mn_2_O_7_)_1−*x*_/(La_1.3_Eu_0.1_Ca_1.6_Mn_2_O_7_)*x* for *μ*_0_*H* = 5 T.

In this context, magnetic properties for La_1.4_Ca_1.6_Mn_2_O_7_ (A) and La_1.3_Eu_0.1_Ca_1.6_Mn_2_O_7_ (B) are described in more detail by means of simulation of Δ*S*_M_(*T*, *μ*_0_*H* = 5 T) of individual compounds La_1.4_Ca_1.6_Mn_2_O_7_ (A) and La_1.3_Eu_0.1_Ca_1.6_Mn_2_O_7_ (B) (see Fig. 5(a)). A numerical method is used to determine the optimum mass ratios of *x* refrigerant samples and the resulting entropy change Δ*S*_M_(*T*, *μ*_0_*H* = 5 T) of the composite.^37^Fig. 5(b) illustrates the numerical calculations for Δ*S*_M_(*T*, *μ*_0_H = 5 T) curves in the vicinity of the magnetic transition temperatures for the investigated materials and their composite. The Δ*S*_M_(*T*, *x*) for (La_1.4_Ca_1.6_Mn_2_O_7_)_1−*x*_/(La_1.3_Eu_0.1_Ca_1.6_Mn_2_O_7_)_*x*_ composites produced by combining a (1 − *x*) × (La_1.4_Ca_1.6_Mn_2_O_7_) and a *x* × (La_1.3_Eu_0.1_Ca_1.6_Mn_2_O_7_) were obtained from their respective Δ*S*_M_(*T*) according to the following equation:1
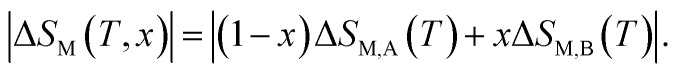
where *x* (0 ≤ *x* ≤ 1) represents the relative weight fraction between A and B compounds. The last equation is used to estimate the |Δ*S*_M_(*T*, *x*)| because it has been demonstrated that the component phases in the solid magnetic refrigerants are mixed without any interactions among themselves; therefore it does not affect the resultant |Δ*S*_M_(*T*, *x*)|.^21^ It is clear from Fig. 5(b) that the numerical Δ*S*_M_(*T*, *x*) represent a double peak resulting from the difference between Curie temperatures Δ*T*_C_ = *T*_CA_ − *T*_CB_ of the constitutive phases for certain values ​​of *x*. We can also see in the same figure that the magnetocaloric responses show a broad single peak for other values ​​of *x* which is clearly obvious near the extreme values ​​*x* = 0 or *x* = 1. The remarkable feature in Fig. 5(b) is that the central region of Δ*S*_M_(*T*, *x*) becomes relatively flat for *x* = 0.48 which shows a table-like behaviour.

To explore the performance of this composite, we have calculated the refrigerant capacity (RC) which is another decisive parameter for evaluating and approving cooling efficiency.^38^ The RC parameter measures the amount of heat convey between the cold and hot reservoirs in the thermodynamic cycle. Thus, it has been suggested as a more suitable indicator of magnetic substances utility for solid-state refrigeration. For practical cooling systems, the RC with a broad temperature range is suitable for the active magnetic refrigeration cycle.^39–41^ The refrigerant capacity depends not only on the maximum of −Δ*S*_M_(*T*), but also on the overall profile of −Δ*S*_M_(*T*). RC is obtained by numerical integration of the area under the −Δ*S*_M_(*T*) curve. The limits of the temperature integration are set by the half-maximum of the Δ*S*_M_(*T*) peak, where *T*_Hot_ and *T*_Cold_ correspond to the two temperatures at which the |Δ*S*_M_(*T*)| value is half of the peak value:^42^2
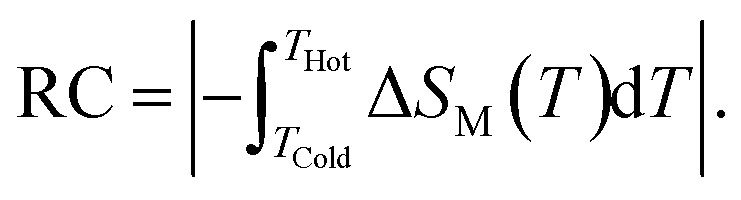


**Fig. 6:**
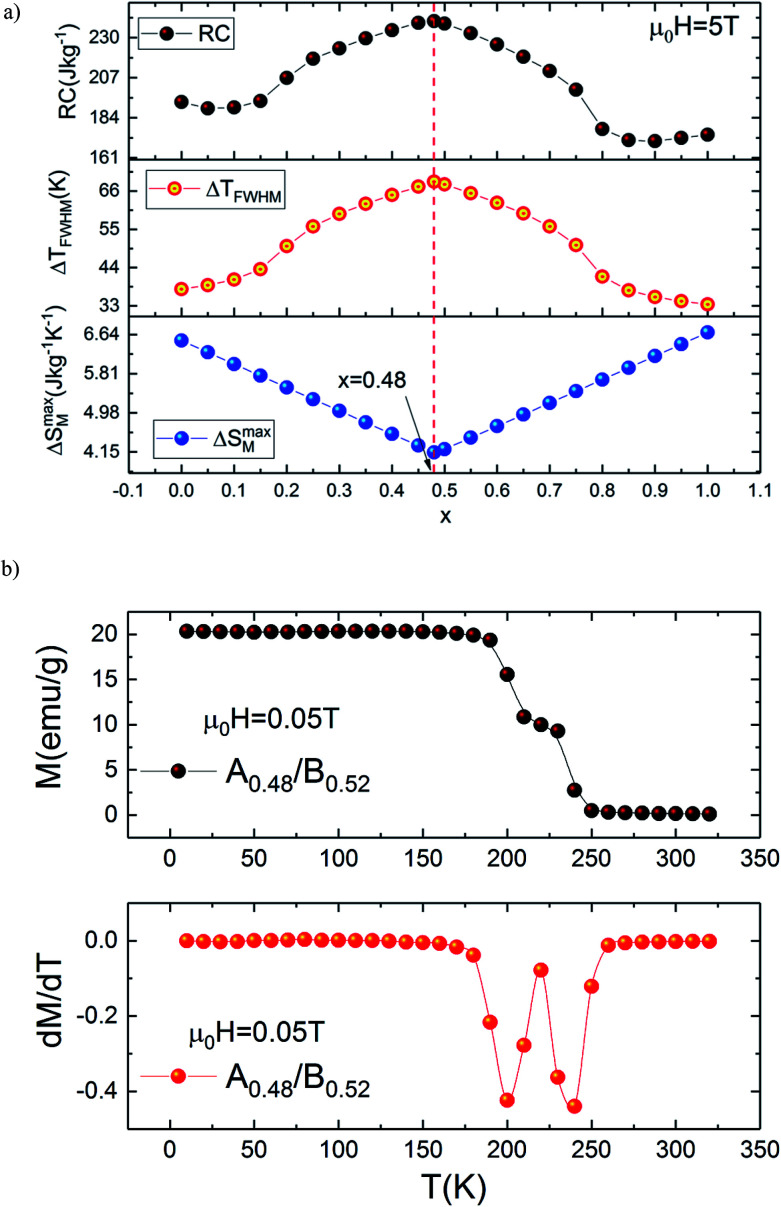
(a) The *x* dependence of Δ*S*^max^_M_, Δ*T*_FWHM_ and the RC for the composites system. (b) (i) Variation of the magnetization *vs.* temperature for the composite (ii) plot of d*M*/d*T* curves as a function of temperature.

Accordingly, we report a detailed investigation of the MCE response as a function of the composite ratio; we present in Fig. 6(a) the *x* dependence of Δ*S*_M_, Δ*T*_FWHM_ and the RC values for the composite system A_*x*_/B_1−*x*_ at *μ*_0_*H* = 5 T. This figure confirms that the important value of Δ*T*_FWHM_ correspond to *x* = 0.48. The existence of the table-like behaviour could give rise to the maximum values ​​of Δ*T*_FWHM_ and RC, which allows the A_0.48_/B_0.52_ composite to become a promising compound for different thermodynamic cycles used in magnetic cooling technology.

Based on these calculations and in order to produce a composite with table-like MCE for a magnetic cooling system using the Ericsson thermodynamic cycle, powdered specimen of the La_1.4_Ca_1.6_Mn_2_O_7_–La_1.3_Eu_0.1_Ca_1.6_Mn_2_O_7_ composite is made by repetitive grinding and mixing of the two compositions in the desired ratio of 48–52% (A_0.48_/B_0.52_). After preparing the composite, the temperature (*T*) dependence of the magnetization (*M*) is measured in field-cooled mode (FC) under an applied field of 0.05 T. The results are depicted in Fig. 6(b) for our new compound. This curve clearly shows that the investigated composite specimen exhibits two magnetic transitions because of its heterogeneous composition. It is also observed in Fig. 6(b) that the pronounced two minima in the d*M*/d*T versus T* curve confirm that the composite contains two magnetic phase transitions compared with individual A and B bilayer manganites. The later successive minima correspond exactly to *T*_C_ for each of the constituent phases A and B used to prepare the A_0.48_/B_0.52_ composite. It is worthwhile to mention that the magnetization magnitude of the studied composite shows a small decrease at low temperatures as compared with that of A and B bilayer manganites. The existence of two transition temperatures originating from different phases can certainly have an important effect on the MCE characteristics because the shape and behaviour of the magnetic entropy change are highly sensitive to the character of the magnetic phase transition.

It is demonstrated that the presence of two magnetic phases in the refrigerant material ensure that the material has a large MCE with a broad refrigeration temperature range and enhanced RC. In this investigation, we used the presence of two magnetic transitions to confirm our above calculation and for generating a broad range of MCE with a significant increase in RC.

To get deeper insight into the magnetocaloric response of the prepared composite upon changing the magnetic field from 0 to 5 T, isothermal magnetization curves of A_0.48_/B_0.52_ are measured as a function of the applied field recorded at different temperatures.

**Fig. 7:**
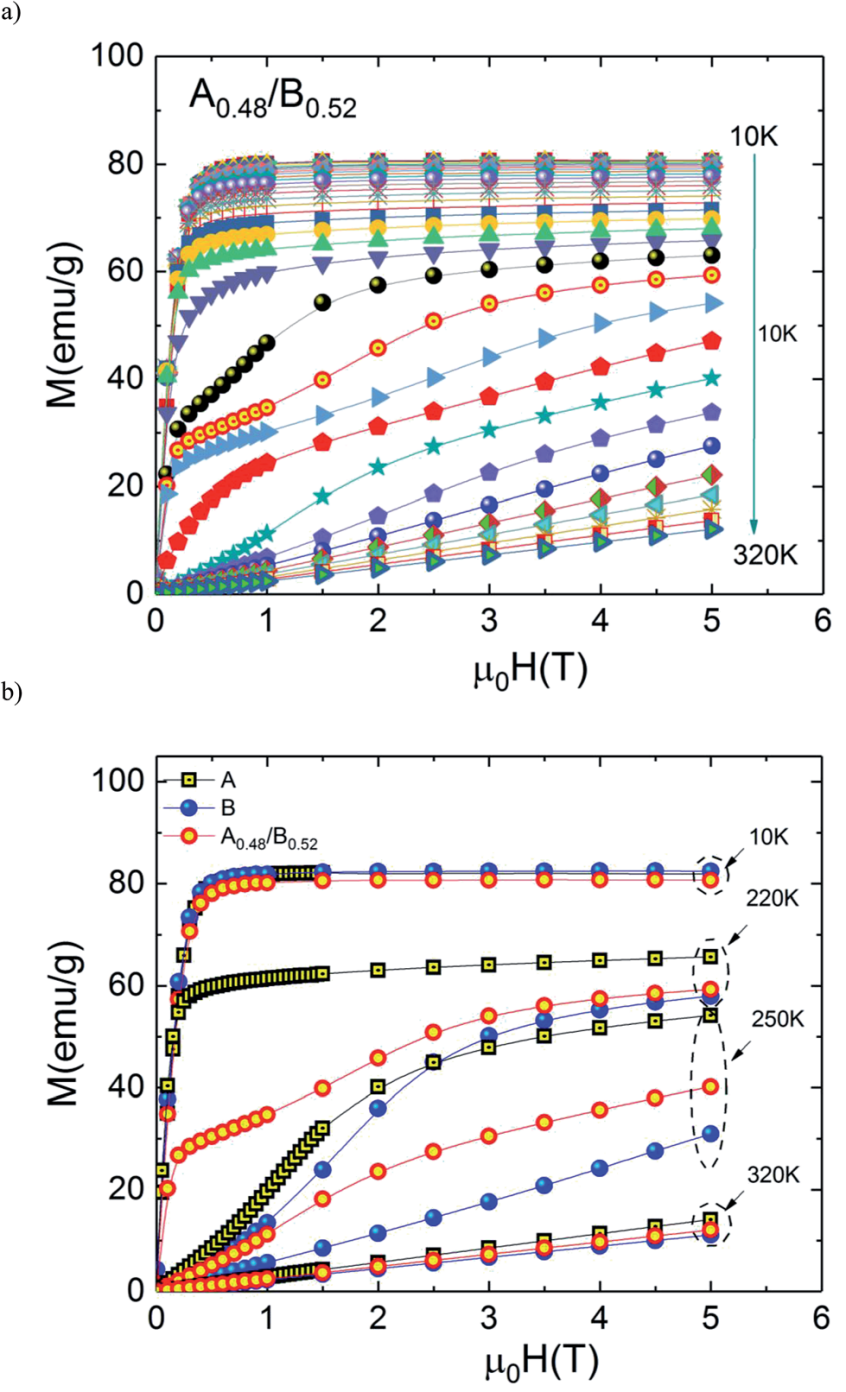
(a) Isothermal magnetization curves at various temperatures for the composite and (b) *M*(*μ*_0_*H*) at 10, 220, 250 and 320 for all samples.

The measured *M*(*μ*_0_*H*) plots are shown in Fig. 7(a). In Fig. 7(b) we compare the selected isothermal *M*–*μ*_0_*H* curves plotted with applied fields between 0 and 5 T at *T* = 10, 220, 250 and 320 K for the individual samples and the A_0.48_/B_0.52_ composite. It is observed from this figure that the A_0.48_/B_0.52_ sample has similar values of magnetization at 10 K and 320 K as compared to A and B samples. In addition, the *M*(*μ*_0_*H*) curves are typical for a ferromagnetic state at 10 K and for a paramagnetic state at 320 K. On the other side, at 220 K and 250 K, the three compounds present different shapes in *M*(*μ*_0_*H*) and the composite system shows the intermediate values of magnetization compared to that of the constituent phases A and B. In this temperature range, the slightly jump in the *M*(*μ*_0_*H*) curves may be attributed to strong domain wall pinning in the ferromagnetic state.

**Fig. 8:**
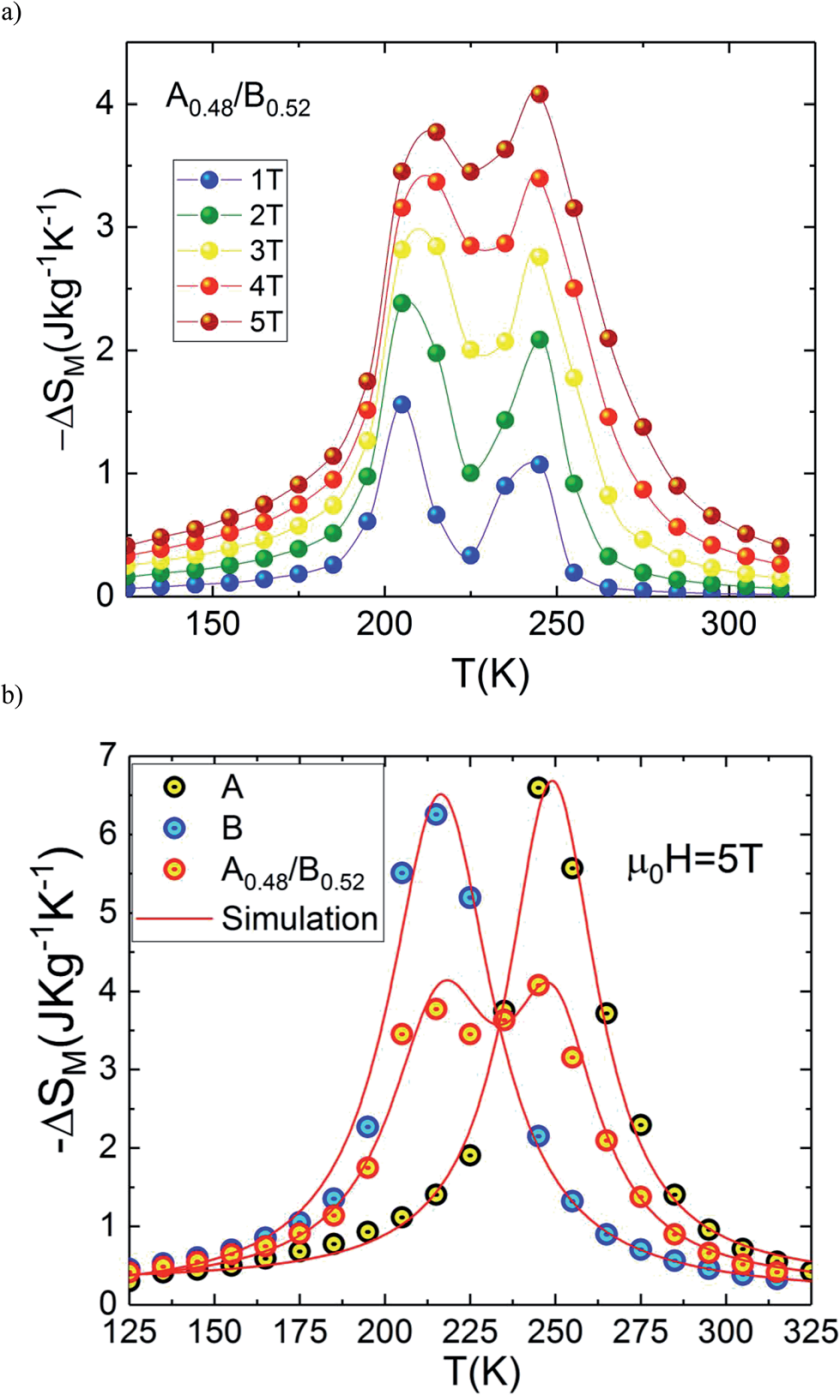
(a) Magnetic entropy change, Δ*S*_M_*vs.* temperature for the composite system and (b) comparison between experimental and simulation.

The temperature dependences of magnetic entropy changes, −Δ*S*_M_(*T*), taken at 1, 2, 3, 4 and 5 T for the A_0.48_/B_0.52_ composite is presented in Fig. 8(a). All the curves of −Δ*S*_M_(*T*) have a clear double-peak shape (two Δ*S*_M_ values), resulting from the disparity in Curie temperature of both phases A and B. The latter double-peak shape is very noticeable at low *μ*_0_*H* and begins to flatten gradually in favor of the table-like behaviour occurring at higher magnetic fields. This behaviour could give rise to the maximum values of Δ*T*_FWHM_ and the RC refrigerant capacity strongly required for the ideal Ericsson cycle magnetic refrigeration over a broad temperature range.^43^

Fig. 8(b) depicts experimental and theoretical entropy change curves for *μ*_0_*H* = 5 T of phases A and B that make up the composite with *T*_C_,_A_ = 200 K and *T*_C,B_ = 240 K, along with Δ*S*_M_(*T*) in the composite *x* = 0.48. Latest figure demonstrates that the agreement between the experimental curves Δ*S*_M_(*T*) and that predicted by eqn (1) is excellent. According to this agreement, we can conclude that the numerical calculations are valid in the choice of MCE composite and can thus be used as means of designing magnetic refrigerant materials with an improved magnetocaloric response for the desired magnetic fields. The maximum value of −Δ*S*_M_(*T*) is found to be 4.07 J kg^−1^ K^−1^ for A_0.48_/B_0.52_ in a wide temperature range. The magnitude of −Δ*S*_M_(*T*) is reduced in the A_0.48_/B_0.52_ composite which gives a broad table-like behaviour with a wide temperature range compared to that of the pure constituent phases. Basically, in an ideal Ericsson cycle, the entropy conveyed between two heat reservoirs (*T*_Hot_ and *T*_cold_) should be as constant as possible to avoid the generation of irreversible work.^44^ For this reason, the flattening of −Δ*S*_M_(*T*, *x* = 0.48) curve can be able to meet the latter requirements for the use of A_0.48_/B_0.52_ as a composite for Ericsson-cycle-based magnetic refrigerators.^43^

**Fig. 9:**
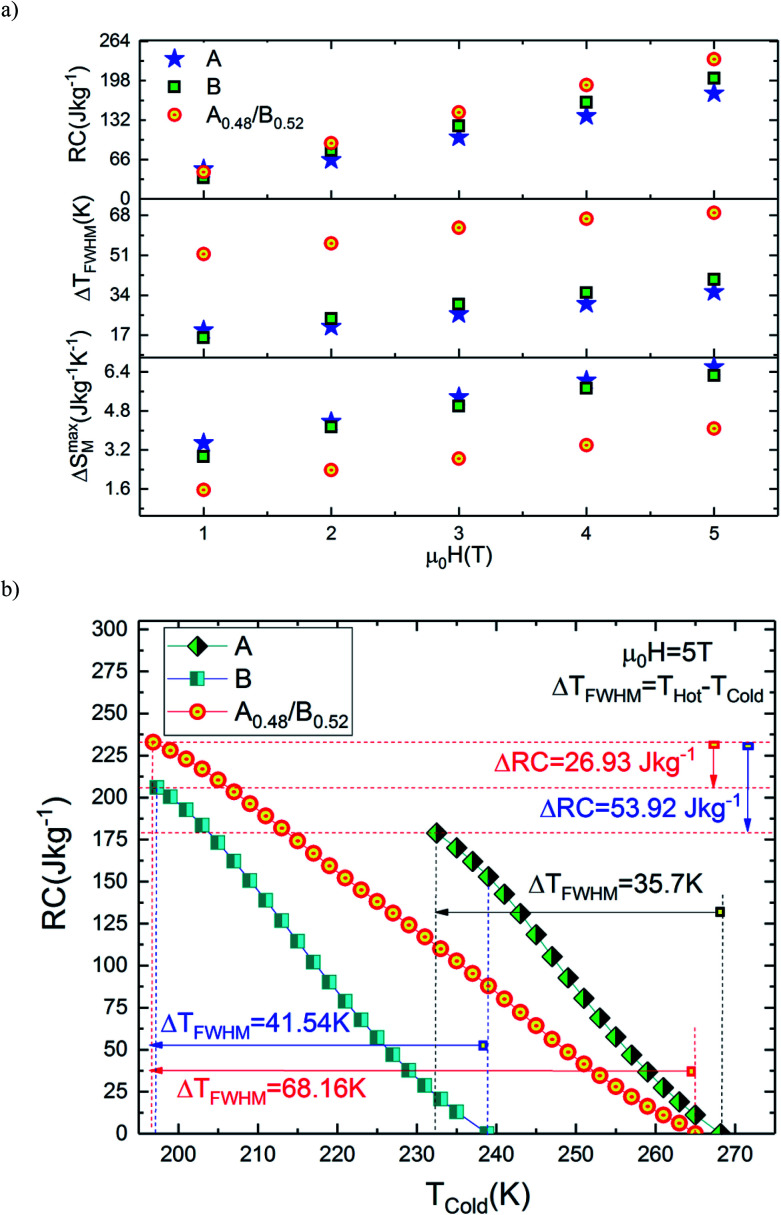
(a) Variation of Δ*S*^max^_M_, Δ*T*_FWHM_ and RC with magnetic field for all samples. (b) RC *versus T*_cold_ for all samples.

From eqn (2), the obtained value of RC is ∼205.92 J kg^−1^ at 5 T in B sample while it does not exceed ∼178.92 J kg^−1^ in A sample which indicate that the Eu-substitution increase the refrigerant capacity. Fig. 9(a) shows Δ*S*^max^_M_, Δ*T*_FWHM_ and RC plots as a function of the applied magnetic field. As displayed in Fig. 9(b), the obtained values of Δ*S*^max^_M_, Δ*T*_FWHM_ and RC are strongly related to the magnetic field. It is clearly observed that the A material has smaller values of Δ*T*_FWHM_ than the B sample. Compared to gadolinium, which is considered as the typical ferromagnetic material for magnetic refrigeration, the RC values of the A and the B samples represents about ∼56.13% and ∼64.6% of the RC estimated for Gd (the value of RC is around 25% lower than that of the relative refrigerant capacity RCP for the Δ*S*_M_(*T*),^42^ from ref. 45 RCP_Gd_ = 425 J kg^−1^ then RC_Gd_ = ¾ × RCP_Gd_ = ¾ × 425 ∼ 319 J kg^−1^). According to the obtained result, the Eu-doped sample is still valuable for magnetic refrigeration at low temperatures. These values are much larger than that of several manganites^46,47^ and are high enough for magnetic cooling. Refrigerants with wide working temperatures and high RC are in fact very beneficial to magnetic cooling applications^48^ and suggests that compounds can thus be used as an active magnetic refrigeration materials suggested by Barclay.^39^ However, in this case the maximum values of Δ*T*_TFWHM_ for all three samples A, B and A_0.48_/B_0.52_ are equal to ∼35.7 K, ∼41.54 K and ∼68.16 K respectively for a field of 5 T. It can be observed that the highest value of Δ*T*_TFWHM_ is revealed for the A_0.48_/B_0.52_ composite and an increment of ∼47.63% and ∼39.05% in Δ*T*_FWHM_ compared with the individual La_1.4_Ca_1.6_Mn_2_O_7_ and La_1.3_Eu_0.1_Ca_1.6_Mn_2_O_7_ samples are observed. This enhancement in Δ*T*_FWHM_ is an outcome of the augmentation of RC despite the diminution of the maximum value of Δ*S*_M_. However, it follows that a compromise is necessary between the value of the Δ*T*_FWHM_ and the energy losses and the efficiency of machine (due to an increase of cycles in the heat exchange medium). The investigated A_0.48_/B_0.52_ composite exhibit nearly constant value of Δ*S*_M_(*T*) with width of ∼68.16 K. The present results confirm that the field and the temperature range used in the numerical calculation are analogous to those explored experimentally and ensure that the large Δ*T*_FWHM_ observed in the prepared composite have a great importance for cooling capacity.

The broadening width of Δ*S*_M_(*T*) is expected to make an increase of RC in the composite as predicted by eqn (2). In this study, it should be noted that the used *T*_Cold_ and *T*_Hot_ are defined as temperatures fulfilling Δ*S*_M_(*T*_Cold_) = Δ*S*_M_(*T*_Hot_) = Δ*S*^max^_M_/2. In order to visualize RC (*T*_Cold_) and obtain the RC values at different values of *T*_Cold_, the integrand is evaluated from high temperature (*T*_Hot_) to low temperature (*T*_Cold_) as depicted in eqn (2). Fig. 9(b) shows the calculated values of RC as a function of *T*_Cold_ under 5 T magnetic field of A, B and A_0.48_/B_0.52_ compounds. It is found that the RC increases as *T*_Cold_ is separate from the Δ*S*_M_ peak temperature (*T*_peak_), at which RC is zero due to eqn (2). In the temperature span of *T*_Hot_ − *T*_Cold_ = Δ*T*_FWHM_, the estimated RC values correspond to A, B and A_0.48_/B_0.52_ samples are 178.92 J kg^−1^, 205.92 J kg^−1^ and 232.85 J kg^−1^ respectively. These values show that the RC of the composite A_0.48_/B_0.52_ is improved by 23.16% and 11.56% when compared with those of A and B samples. This proves the superior cooling power of the A_0.48_/B_0.52_ sample and refers to a possible way to optimize RC of refrigerant magnetic materials. This result reveals that the A_0.48_/B_0.52_ is a promising compound to be used as a refrigerant material in solid-state refrigeration with the Ericsson cycle. Similar reinforcement in RC responses are observed in many composite systems like La_1.4_Ca_1.6_Mn_2_O_7_/(La_0.08_Gd_0.02_)_1.4_Ca_1.6_Mn_2_O_7_,^49^ FeZrB(Cu),^50^ and La_0.7_Ca_0.3_Mn^16^O_3_/La_0.7_Ca_0.3_Mn^18^O_3_.^51^

To make our analysis more complete, we are concerned with the nature of the magnetic phase transition in our bilayer manganites. For that reason, we have investigated the field dependence of MCE in A, B and A_0.48_/B_0.52_ samples by using the relation expressed as Δ*S*_M_ ≈ *a*(*μ*_0_*H*)^*N*^. The latter relationship makes it possible to determine the nature of the magnetic phase transition observed in the aforementioned samples. Recently, J. Y. Law *et al.* proposed a quantitative criterion to identify the order of magnetic phase transitions using the field dependence of magnetocaloric effect. For materials with the first-order magnetic phase transition (FOPT), Δ*S*_M_ depends on the field with exponent *N* > 2.^52^ Particularly, for magnetic samples, the local exponent *N*(*T*,*μ*_0_*H*) can be calculated from the logarithmic derivative of the experimental Δ*S*_M_(*T*, *μ*_0_*H*):^53^3
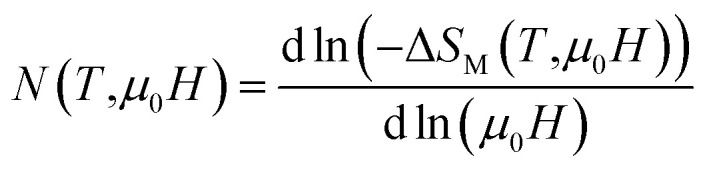


**Fig. 10:**
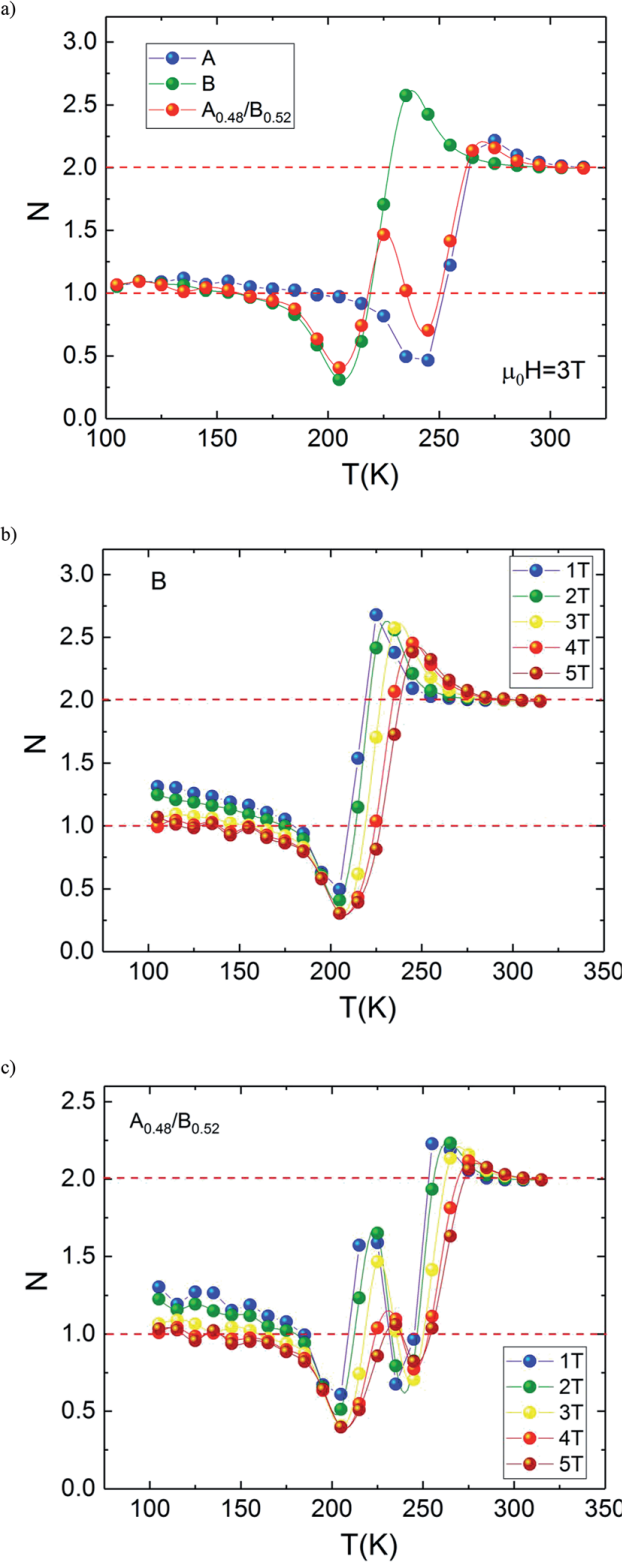
Temperature dependence of the field dependence exponent *N* for (a) La_1.3_Eu_0.1_Ca_1.6_Mn_2_O_7_, (b) composite. (c) Temperature dependence of the field dependent exponent *N* (at *μ*_0_*H* = 3 T) for the La_1.4_Ca_1.6_Mn_2_O_7_, La_1.3_Eu_0.1_Ca_1.6_Mn_2_O_7_ and the composite.

**Fig. 11:**
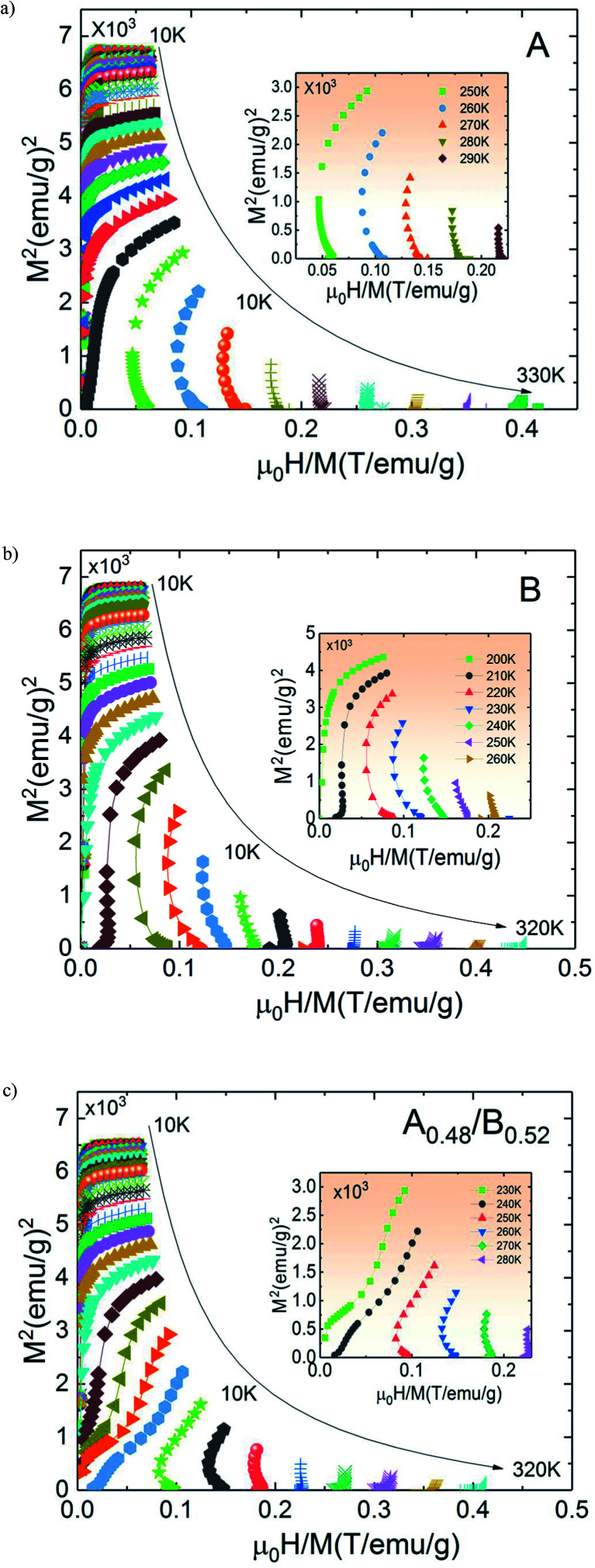
Arrott curves for (a) La_1.4_Ca_1.6_Mn_2_O_7_ (b) La_1.3_Eu_0.1_Ca_1.6_Mn_2_O_7_ and (c) the composite.

The temperature dependence of *N* is illustrated in Fig. 10(a) for A, B and A_0.48_/B_0.52_ samples. The exponent *N*, for A and B samples has a minimum value at *T*_C_. However, for A_0.48_/B_0.52,_ the *N*(*T*) curves exhibits two minima whose positions are related to the critical temperatures of the existing phases (A and B) in this composite. On the one hand, it is observed in Fig. 10 that the exponent *N* is sensitive to the magnetic field in the entire studied temperature range and the magnetic entropy changes, Δ*S*_M_. The value of *N*(*T*_C_) < 0.4 at high magnetic fields indicates that our samples undergo first order magnetic transition temperature.^54,55^ In the other hand and under critical temperatures, the *N*(*T*) curves increase gradually with the temperature drop and approaches 1 for higher magnetic fields. Far above *T*_C_, the *N*(*T*) values overshoots 2 (*N* > 2) in the paramagnetic region (near magnetic transitions) of all three samples. This overshoot is more pronounced in sample B compared to sample A and the composite A_0.48_/B_0.52_. The observed behaviour shows that the quantitative criterion of *N* > 2 near the transition is valid for monophasic and biphasic materials which indicates that our samples exhibit a first-order transition. This is in agreement with the previous observations in *N*(*T*_C_) values. A similar behaviour is reported in other magnetic materials with first-order transition.^56^ However, the order of magnetic phase transition is usually revealed by the Arrott plots (*M*^2^*vs. H*/*M*). For more confirmation of the nature of the magnetic phase transition of A, B and A_0.48_/A_0.52_ samples, the curves of *M*^2^*vs. H*/*M* plotted at different temperatures are exhibited in Fig. 11. The Arrott plots for the aforementioned materials just above the respective *T*_C_ are displayed in the inset of the Fig. 11. According to the Banerjee criterion, the obviously negative slopes of Arrott plots verify the first-order nature of the three samples,^57,58^ which is consistent with the quantitative criterion.

## 4. Conclusion

In summary, we have first investigated the structural, magnetic and magnetocaloric properties of La_1.4_Ca_1.6_Mn_2_O_7_ and La_1.3_Eu_0.1_Ca_1.6_Mn_2_O_7_ samples prepared by the standard solid-state reaction method. The magnetic study showed that our investigated samples exhibit a PM–FM transition and present large magnetocaloric properties. Secondly, we have prepared a composite using the aforementioned samples with weight ratio of 48–52%. The latter ratio is determined numerically to obtained high magnetocaloric performances. Compared with the main polycrystalline phases, the magnetic entropy change of the prepared composite was found to be smaller. The prepared composite is characterized by important values of Δ*T*_FWHM_ (68.16 K under 5 T) and RC (232.85 J kg^−1^ under 5 T). The refrigeration capacity of the composite is enhanced by 23.16% and 11.56% when compared with those of the individual La_1.4_Ca_1.6_Mn_2_O_7_ and La_1.3_Eu_0.1_Ca_1.6_Mn_2_O_7_ samples. Hence, the results of the A_0.48_/B_0.52_ composite represent a significant motivation to search for new suitable magnetic material with several reversible magnetic transitions originating from two different phases in order to expand working temperature with the same sign of successive magnetic entropy changes. These observations corroborate that the magnetic refrigerant compound with the more competitive characteristics may be developed in a form of a composite material that can lead to a future cheaper, more efficient and green refrigerator. In addition, our magnetocaloric investigation shows that the first order phase transition is observed on our compounds. We showed that using the field dependence of magnetocaloric effect, the order of the phase transition can be unambiguously determined using a quantitative criterion even for *N* > 2 near the transition of monophasic and biphasic magnetocaloric materials. The order of the magnetic phase transition of the three samples is corroborate by using the Banerjee criterion.

## Conflicts of interest

There are no conflicts of interest to declare.

## Supplementary Material
